# The effect of transferring a poor-quality embryo together with a good-quality embryo on the singleton birth weight: A retrospective cohort study

**DOI:** 10.18502/ijrm.v20i2.10500

**Published:** 2022-03-21

**Authors:** Saeideh Dashti, Atefeh Pejman, Nasim Tabibnejad, Maedeh Mortezanasab

**Affiliations:** Research and Clinical Center for Infertility, Yazd Reproductive Sciences Institute, Shahid Sadoughi University of Medical Sciences, Yazd, Iran.

**Keywords:** Embryo quality, Birth weight, Frozen-embryo transfer, Fresh embryo transfer, Single embryo transfer, Double embryo transfer.

## Abstract

**Background:**

Embryo quality may affect birth weight among neonates born through assisted reproductive technology. There are very limited studies assessing the adverse effect of transferring a poor-quality embryo with a good-quality one on neonatal outcomes.

**Objective:**

The aim of this study was to evaluate the effect of double embryo transfer (DET) with one good-quality embryo (GQE) plus a poor-quality one on the birth weight of newborns conceived by in vitro fertilization in both fresh and frozen-thawed embryo transfer cycles.

**Materials and Methods:**

This study was conducted at Yazd Reproductive Sciences Institute, Yazd, Iran. A total of 626 women were classified into three groups according to the embryo quality: single embryo transfer with a GQE (group A); DET using two GQEs (group B); and DET using one good-quality and one poor-quality embryo (group C). The primary outcome was singleton birth weight which was compared between the three groups among fresh and frozen-embryo transfer cycles. A comparative analysis was also performed regarding the effect of vitrification procedures on neonatal birth weight within each of the three embryo quality-based groups.

**Results:**

The mean birth weight and the rate of preterm birth were similar between the three groups (p = 0.45 and 0.32, respectively). There were also no significant differences found in the vitrification comparative analysis between and within the groups with regard to birth weight.

**Conclusion:**

Our results showed that a poor-quality embryo did not have a significant influence on a good-quality one regarding neonatal birth weight when transferred together.

## 1. Introduction

In vitro fertilization (IVF) has some unknown features which may adversely affect IVF-born babies. The risk of low birth weight (LBW), preterm birth (PTB), and being small for gestational age are increased among neonates born through assisted reproductive technology (ART) cycles in comparison with naturally conceived ones (1). Moreover, the laboratory procedures, including the method of insemination, culture medium and duration of culture period, influence the embryo quality, which has been shown to have a critical role in successful pregnancy (2, 3).

Even though transferring good-quality embryos (GQEs) is preferable, for a fairly large number of patients without GQEs, the transfer of poor-quality embryos (PQEs) is unavoidable. It has been previously reported that double embryo transfer (DET) may increase ongoing pregnancy and live birth rates and therefore, some IVF clinics transfer more than one embryo. In using the DET strategy with only one GQE available, the second embryo must be a PQE. There are very limited studies assessing the adverse effect of an additional PQE on the neonatal outcomes through DET.

On the other hand, frozen-thawed embryo transfer (FET) has become a common ART procedure and so determining the effect of cryopreservation on embryo quality is crucial. In addition, it has been indicated that cryopreservation is strongly involved in the variation of neonatal birth weight (4, 5). As LBW has been associated with the incidence of serious complications such as cardiovascular diseases, hypertension and Type 2 diabetes (6), it is important to know whether the combination of one GQE and one PQE transferred in a DET program might affect neonatal birth weight in both fresh and FET cycles. Thus, the aim of the current study was to evaluate the effect of DET with one GQE plus one PQE on the birth weight of newborns conceived by ART in both fresh and FET cycles.

## 2. Materials and Methods 

### Study population and design

This retrospective cohort study assessing the effect of embryo quality on the neonatal birth weight after transfer of cleavage-stage embryos in both fresh and FET cycles was conducted at Yazd Reproductive Sciences Institute in Yazd, Iran. The data of 626 infertile women aged 20 to 44 yr, who had undergone IVF/intracytoplasmic sperm injection (ICSI) cycles and delivered an alive singleton birth, were consecutively reviewed between July 2017 and September 2021. The women who planned to have one or two cleavage-stage embryos transferred were included in our study. The data were extracted from hospital electronic medical records and only women with complete hospital records were included. The exclusion criteria were body mass index (BMI) ≥ 30 kg/m^2^, uterine anomalies, gestational diabetes, pregnancy-induced hypertension, preeclampsia and uncontrolled endocrine or immune disorders. Women with oocyte donation or surrogate cycles, women with preimplantation genetic testing or couples with severe male factor infertility were also excluded from all analyses. Women were enrolled only once in the study and if women had more than one treatment cycle during the study period, only the first cycle was considered.

### Ovarian stimulation and oocyte retrieval

Women were stimulated with gonadotropin releasing hormone (GnRH) agonist, GnRH antagonist, or microdose flare protocols. In the GnRH agonist long protocol, 0.1 mg triptorelin acetate (Decapeptyl, Ferring Pharmaceuticals, the Netherlands) was injected subcutaneously each day from the 21 ${}^{\text{st}}$ day of the menstrual cycle. The dose of decapeptyl was reduced to 0.05 mg on the 2 ${}^{\text{nd}}$ day of the menstrual cycle and ovarian stimulation was commenced with a subcutaneous dose of 150-225 IU recombinant human follicle stimulating hormone (rFSH) (Cinnal-f, Cinnagen, Iran) once a day.

For women who were stimulated by the GnRH antagonist protocol, the starting dose of gonadotropin was adjusted in accordance with their ages, Anti-Mullerian hormone (AMH) level, antral follicle count, and ovarian response, which was assessed using serial transvaginal ultrasonography during ovarian stimulation. From 150 to 300 IU/day rFSH was administered subcutaneously for five days starting on cycle day two. Once a leading follicle ≥ 14 mm was observed in ultrasonography, 0.25 mg per day of subcutaneous GnRH antagonist (Cetrotide, Merck Serono, Aubonne, Switzerland) was injected.

In the microdose flare protocol, buserelin (CinnaFact, Cinnagen, Iran) was administered at the dose of 0.05 mg subcutaneously twice daily starting on cycle day two, which continued until the day of human chorionic gonadotropin (hCG) injection. Ovarian stimulation was started using a daily subcutaneous injection of 225 to 300 IU rFSH on cycle day four. Oocyte triggering was done when at least two follicles reached 17 mm in diameter in ultrasonography. Oocyte triggering was performed using either 10,000 IU hCG (Pregnyl, Organon, the Netherlands) or 0.2 mg of subcutaneous GnRH-a (Decapeptyl, Ferring Pharmaceuticals, the Netherlands). However, in some cases, oocyte triggering was done using a dual-trigger method by administration of 1500-5000 IU hCG plus 0.2 mg of subcutaneous GnRH-a.

Transvaginal ultrasound-guided oocyte retrieval was carried out 36 hr after hCG administration for all subjects. Oocytes were inseminated using conventional IVF or ICSI as standard protocols.

### Embryo grading, vitrification and warming

Cleavage-stage embryos were graded according to the number of blastomeres and fragmentation degree from A (the best quality) to D (the worst quality). Grade A: embryos with 7-9 blastomeres and a maximum of 20% cytoplasmic fragmentation; Grade B: embryos with 7-9 blastomeres and over 20% fragmentation; Grade C: embryos with 4-6 blastomeres and a maximum of 20% fragmentation; Grade D: embryos with 4-6 blastomeres and over 20% fragmentation (7). Embryo grading was done by a team of three highly experienced embryologists. In this study, we considered grade A and B embryos as good quality if they possessed 7-9 blastomeres on day three and contained ≤ 20% fragmentation. The embryos of grade C and D were defined as poor quality if the embryos had 4-6 blastomeres on day three and ≥ 20% fragmentation. In the single embryo transfer (SET) GQE group, the transferred embryo met the criteria for good quality (group A). Likewise, in the DET-GQE group, the two transferred embryos met the criteria for good quality (group B). In group C, one of the transferred embryos met the criteria for good quality and the other one met the criteria for poor quality.

Embryo vitrification and warming were performed as previously described (8, 9). In short, embryos containing less than 30% fragmentation were cryopreserved via the vitrification method (9). Thawing was carried out at least two months after cryopreservation based on the VitroLife protocol (VitroLife, Gothenburg, Sweden) (8). Cryopreserved-thawed embryos with ≥ 50% intact blastomeres and without damaged zona pellucida were considered to have survived morphologically.

### Embryo transfer

The best quality embryos were selected for fresh embryo transfer 48-72 hr after oocyte retrieval. Routinely, a maximum of two fresh or vitrified-warmed embryos of good quality were transferred using an embryo transfer Labotect catheter (Labor-Technik-Göttingen GmbH, Gottingen, Germany) in the fresh and FET group, respectively. In some cases, one GQE and one PQE were transferred as there was no alternative.

### Endometrial preparation for FET cycles

For endometrial preparation, 6 mg per day oral estradiol valerate (Estradiol Valerate, Aburaihan CO, Iran) was administered starting on cycle day two until the endometrial thickness reached 7.5 mm. After that, all women received 400 mg of vaginal cyclogest pessaries (Cox Pharmaceuticals, Barnstaple, UK) twice daily. Both estradiol and progesterone were administered until observation of fetal heart activity by ultrasound.

### Ethical considerations

This study was approved by the Ethics Committee of Yazd Reproductive Sciences Institute, Shahid Sadoughi University of Medical Sciences, Yazd, Iran (Code: IR.SSU.RSI.REC.1399.043).

### Statistical analysis

The SPSS software (Statistical Package for the Social Sciences, version 25, Chicago, IL, USA) was used for data analysis. The primary outcome was singleton birth weight. The other neonatal factors, as secondary outcomes, included gestational age, LBW (birth weight < 2500 gr), very LBW (birth weight < 1500 gr), high birth weight (birth weight > 4500 gr), and PTB (delivery < 37 gestational wk). The Kolmogorov-Smirnov test did not show a normal distribution for all continuous variables. The baseline characteristics and neonatal outcomes were compared between the three groups using the Kruskal-Wallis test for continuous data and the Chi-square test for categorical data. Multivariable logistic regression analysis was run to find the association between embryo quality, and birth weight and preterm delivery. The following confounders were entered in the multivariable analyses: maternal age and BMI, paternal age, parity, gravidity, infertility type, cause and duration, insemination method, type of ovarian stimulation, concentrations of AMH and estradiol, number of retrieved and Metaphase II oocytes and 2 pronuclears (2PNs), endometrial thickness, fresh or FET cycles, delivery method and newborn gender. P-values < 0.05 were considered significant.

## 3. Results

**Figure 1:**
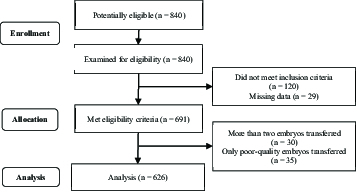
Flow-chart of the study population, enrollment, allocation and analysis.

A total of 840 infertile women who had undergone IVF/ICSI cycles and delivered an alive singleton birth were identified to participate in the study and were assessed for the eligibility criteria. Of those, 720 women met the inclusion criteria and 29 women were excluded because of missing data. Thirty women were excluded due to transferring more than two embryos and 35 were excluded due to transferring only PQEs. Finally, the data of 626 women were extracted for analysis (Figure 1), of which, 258 women were in the fresh embryo transfer group, and FET was performed for 368 women. The patients' basic characteristics and ART cycle features along with neonatal outcomes were compared according to the quality of the transferred embryos in both the fresh and FET cycles. The demographic and treatment cycle characteristics in the fresh and FET groups are listed in table I and II, respectively. No differences were observed in terms of maternal or paternal age, maternal BMI, parity, gravidity, infertility cause or duration, insemination method, type of ovarian stimulation, or endometrial thickness between the groups. Nevertheless, the number of retrieved and mature oocytes and the number of 2PNs differed significantly among the embryo quality categories in the fresh group (p < 0.001). In addition, the AMH and E2 concentrations as well as the number of 2PNs were significantly different between the groups with different embryo quality classifications in the FET group.

The neonatal outcomes categorized by embryo quality in the fresh and FET groups are presented in table III and table IV, respectively. The mean birth weight and the rate of preterm birth were similar between the three groups regarding embryo quality.

**Figure 2:**
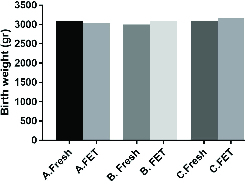
Neonatal birth weight according to the embryo quality in both fresh and frozen embryo transfer cycles. A: Group A, B: Group B, C: Group C.

In addition, a comparative analysis was performed regarding the effect of vitrification procedures on neonatal birth weight within each of the three embryo quality-based groups — group A: SET using one GQE (this included 37 fresh and 50 FET cycles); group B: DET using two GQEs (this included 181 fresh and 244 FET cycles); and group C: DET using one GQE plus one PQE (this included 40 fresh and 74 FET cycles). There were no significant differences between and within the groups with regard to birth weight (Figure 2).

To adjust for the confounding factors, we carried out a logistic regression analysis to investigate the relationship between embryo quality, and birth weight and preterm delivery. No association was found between the embryo quality and birth weight and preterm delivery (Table V).

**Table 1:** Women's demographic and treatment cycle characteristics according to embryo quality in fresh cycles


**Variable**		**Group A (n = 37)**	**Group B (n = 181)**	**Group C (n = 40)**	**p-value**
Maternal age (yr)*		32.0 (8)	31.5 (7)	31.5 (8)	0.66
Maternal BMI (kg/m^2^)*		24.6 (5.5)	25.2 (4.4)	25.7 (3.9)	0.78
Paternal age (yr)*		36.0 (6)	36.0 (6)	37.0 (6)	0.79
Parity**					
	**0** * *	31 (83.8)	154 (85.1)	36 (90.0)	0.68
	**≥ 1**	6 (16.2)	27 (14.9)	4 (10.0)	
Gravidity**					
* *	**0** * *	28 (75.7)	133 (73.5)	30 (75.0)	0.77
	**1**	6 (16.2)	28 (15.5)	8 (20.0)	
	**≥ 2**	3 (8.1)	20 (11.0)	2 (5.0)	
Infertility type**					
* *	**Primary** * *	28 (75.7)	133 (73.5)	30 (75.0)	0.95
	**Secondary**	9 (24.3)	48 (26.5)	10 (25.0)	
Infertility duration (yr)* * *		4.0 (6)	5.0 (5)	4.0 (5)	0.41
Infertility cause**					
* *	**Male** * *	7 (18.9)	50 (27.6)	15 (37.5)	0.05
	**PCOS**	5 (13.5)	18 (9.9)	1 (2.5)	
	**Tubal factor**	1 (2.7)	1 (0.6)	1 (2.5)	
	**Endometriosis**	3 (8.1)	4 (2.2)	5 (12.5)	
	**Diminished ovarian reserve**	6 (16.2)	30 (16.6)	9 (22.5)	
	**Mixed**	12 (32.4)	53 (29.3)	6 (15.0)	
	**Unexplained**	3 (8.1)	25 (13.8)	3 (7.5)	
Fertilization method**					
* *	**IVF** * *	7 (18.9)	41 (22.7)	9 (22.5)	0.43
	**ICSI**	25 (67.6)	128 (70.7)	30 (75.0)	
	**Mixed**	5 (13.5)	12 (6.6)	1 (2.5)	
Ovarian stimulation protocol**					
* *	**GnRH agonist** * *	5 (13.5)	20 (11.0)	5 (12.5)	0.76
	**GnRH antagonist**	28 (75.7)	137 (75.7)	27 (67.5)	
	**Microdose flare**	4 (10.8)	24 (13.3)	8 (20.0)	
AMH (ng/ml)* * *		2.0 (3.5)	2.4 (2.8)	2.1 (1.8)	0.35
Estradiol on the day of trigger (pg/ml)*		897.0 (961)	1148.0 (954)	1128.0 (739)	0.12
Number of retrieved oocytes*		4 (4)	7 (5)	7 (5)	< 0.001
Number of MII oocytes*		3 (4)	6 (5)	6 (4)	< 0.001
Number of 2PNs*		1 (0)	4 (2)	3 (4)	< 0.001
Endometrial thickness (mm)*		9.3 (2.5)	10.0 (2.3)	9.0 (3.0)	0.08
Data are presented as *Median (Interquartile range) and **Number (%). Group A: Single embryo transfer using a good-quality embryo, Group B: Double embryo transfer using two good-quality embryos, Group C: Double embryo transfer using one good-quality embryo plus one poor-quality embryo, *Analysis using Kruskal-Wallis test, **Analysis using Chi-square test, BMI: Body mass index, PCOS: Polycystic ovarian syndrome, IVF: In vitro fertilization, ICSI: Intracytoplasmic sperm injection, GnRH: Gonadotropin releasing hormone, AMH: Anti-Mullerian hormone, MII: Metaphase II, 2PN: 2 pronuclear					

**Table 2:** Women's demographic and treatment cycle characteristics according to embryo quality in FET cycles


**Variable**		**Group A (n = 50)**	**Group B (n = 244)**	**Group C (n = 74)**	**p-value**
Maternal age (yr)*		33.0 (10)	32.0 (7)	33.0 (6)	0.41
Maternal BMI (kg/m^2^)*		24.6 (3.8)	24.5 (3.6)	24.9 (3.6)	0.79
Paternal age (yr)*		37.0 (7)	35.0 (7)	36.0 (7)	0.06
Parity**					
* *	**0** * *	43 (86.0)	223 (91.4)	71 (95.9)	0.14
	** ≥ 1**	7 (14.0)	21 (8.6)	3 (4.1)	
Gravidity**					
* *	**0** * *	39 (78.0)	185 (75.8)	62 (83.8)	0.55
	**1**	6 (12.0)	39 (16.0)	9 (12.2)	
	** ≥ 2**	5 (10.0)	20 (8.2)	3 (4.1)	
Infertility type**					
* *	**Primary** * *	39 (78.0)	185 (75.8)	62 (83.8)	0.35
	**Secondary**	11 (22.0)	59 (24.2)	12 (16.2)	
Infertility duration (yr)* * *		6 (5.0)	5 (5.0)	5.5 (5.2)	0.76
Infertility cause**					
* *	**Male** * *	15 (30.0)	54 (22.1)	17 (23.0)	0.40
	**PCOS**	11 (22.0)	66 (27.0)	20 (27.0)	
	**Tubal factor**	1 (2.0)	3 (1.2)	3 (4.1)	
	**Endometriosis**	0 (0)	8 (3.3)	0 (0)	
	**Diminished ovarian reserve**	4 (8.0)	20 (8.2)	2 (2.7)	
	**Mixed**	11 (22.0)	31 (12.7)	8 (10.8)	
	**Unexplained**	8 (16.0)	62 (25.4)	24 (32.4)	
Fertilization method**					
* *	**IVF** * *	4 (8.0)	54 (22.1)	14 (18.9)	0.24
	**ICSI**	39 (78.0)	157 (64.3)	49 (66.2)	
	**Mixed**	7 (14.0)	33 (13.5)	11 (14.9)	
Ovarian stimulation protocol**					
* *	**GnRH agonist** * *	6 (12.0)	32 (13.1)	4 (5.4)	0.09
	**GnRH antagonist**	38 (76.0)	195 (79.9)	68 (91.9)	
	**Microdose flare**	6 (12.0)	17 (7.0)	2 (2.7)	
AMH (ng/ml)* * *		3.2 (4.3)	3.9 (4.6)	5.3 (5.3)	0.01
Estradiol on the day of trigger (pg/ml)*		1602 (1540)	2108 (1964)	2283 (1919)	0.04
Number of retrieved oocytes*		13 (14)	14 (11)	15 (14)	0.10
Number of MII oocytes*		11 (9)	11 (9)	11.5 (12)	0.14
Number of 2PNs*		5 (9)	7 (7)	8 (6)	< 0.01
Endometrial thickness (mm)*		9.4 (2.5)	9.5 (2.1)	9.5 (2.7)	0.67
Data are presented as *Median (Interquartile range) and **Number (%). Group A: Single embryo transfer using a good-quality embryo, Group B: Double embryo transfer using two good-quality embryos, Group C: Double embryo transfer using one good-quality embryo plus one poor-quality embryo, *Analysis using Kruskal-Wallis test, **Analysis using Chi-square test, BMI: Body mass index, PCOS: Polycystic ovarian syndrome, IVF: In vitro fertilization, ICSI: Intracytoplasmic sperm injection, GnRH: Gonadotropin releasing hormone, AMH: Anti-Mullerian hormone, MII: Metaphase II, 2PN: 2 pronuclear, FET: Frozen-thawed embryo transfer					

**Table 3:** Neonatal outcomes of live born singletons by embryo quality in fresh cycles


**Variable**		**Group A (n = 37)**	**Group B (n = 181)**	**Group C (n = 40)**	**p-value**
**Newborn gender**					
	**Female**	17 (45.9)	95 (52.5)	17 (42.5)	0.45
	**Male**	20 (54.1)	86 (47.5)	23 (57.5)	
**Method of delivery**					
	**NVD**	7 (18.9)	32 (17.7)	4 (10.0)	0.46
	**C/S**	30 (81.1)	149 (82.3)	36 (90.0)	
**Gestational age (wk)**					
	** < 37**	5 (13.5)	30 (16.6)	6 (15.0)	0.88
	** ≥ 37**	32 (86.5)	151 (83.4)	34 (85.0)	
**Birth weight (gr)**					
	**Median (IQR)**	3100 (538)	3000 (600)	3100 (660)	0.13
	**Very low birth weight ( < 1500)**	2 (5.4)	7 (3.9)	0 (0)	0.49
	**Low birth weight (1500-2500)**	2 (5.4)	22 (12.2)	4 (10.0)	
	**High birth weight ( > 4500)**	0	0	0	
Data are presented as number (%). Group A: Single embryo transfer using a good-quality embryo, Group B: Double embryo transfer using two good-quality embryos, Group C: Double embryo transfer using one good-quality embryo plus one poor-quality embryo, Analysis using Chi-square test, NVD: Normal vaginal delivery, C/S: Cesarean section, IQR: Interquartile range					

**Table 4:** Neonatal outcomes of live born singletons by embryo quality in FET cycles


**Variable**		**Group A (n = 50)**	**Group B (n = 244)**	**Group C (n = 74)**	**p-value**
**Newborn gender**					
	**Female**	21 (42.0)	137 (56.1)	33 (44.6)	0.07
	**Male**	29 (58.0)	107 (43.9)	41 (55.4)	
**Method of delivery**					
	**NVD**	8 (16.0)	29 (11.9)	9 (12.2)	0.72
	**C/S**	42 (84.0)	215 (88.1)	65 (87.8)	
**Gestational age (wk) **					
	** < 37**	5 (10.0)	38 (15.6)	7 (9.5)	0.29
	** ≥ 37**	45 (90.0)	206 (84.4)	67 (90.5)	
**Birth weight (gr)**					
	**Median (IQR)**	3025 (586)	3100 (695)	3100 (463)	0.95
	**Very low birth weight ( < 1500)**	1 (2.0)	8 (3.3)	0 (0)	0.47
	**Low birth weight (1500-2500)**	5 (10.0)	21 (8.6)	5 (6.8)	
	**High birth weight ( > 4500)**	1 (2.0)	1 (0.4)	0 (0)	
Data are presented as number (%). Group A: Single embryo transfer using a good-quality embryo, Group B: Double embryo transfer using two good-quality embryos, Group C: Double embryo transfer using one good-quality embryo plus one poor-quality embryo. Analysis using Chi-square test, NVD: Normal vaginal delivery, C/S: Cesarean section, IQR: Interquartile range, FET: Frozen-thawed embryo transfer					

**Table 5:** Results of multiple regression analysis of singleton birth weight and preterm delivery


**Variable**		**Birth weight**		**Preterm delivery**	
		**OR (95% CI)**	**p-value**	**OR (95% CI)**	**p-value**
**Maternal age (yr)**		0.99 (0.92-1.06)	0.86	1.04 (0.97-1.11)	0.19
**Maternal BMI (kg/m^2^)**		1.05 (0.95-1.15)	0.29	1.05 (0.96-1.15)	0.24
**Paternal age (yr)**		1.03 (0.96-1.10)	0.34	0.95 (0.89-1.01)	0.16
**Parity (0 vs. ≥ 1)**		0.95 (0.33-2.76)	0.93	1.49 (0.55-4.05)	0.43
**Gravidity**					
* *	**0** * *	Ref	Ref	0.83
	**1**	0.99 (0.32-3.07)	0.10		1.12 (0.39-3.23)
**Infertility type (primary vs. secondary)** * *		1.09 (0.33-3.56)	0.88	0.70 (0.23-2.16)	0.54
**Infertility duration (yr)**		1.01 (0.94-1.10)	0.62	1.01 (0.94-1.08)	0.70
**Infertility cause**					
* *	**Male** * *	Ref	Ref	
	**PCOS**	1.60 (0.61-4.19)	0.34	0.50 (0.19-1.31)	0.16
	**Tubal factor **	1.04 (0.11-9.93)	0.97	0.92 (0.10-8.23)	0.95
	**Endometriosis **	0.80 (0.21-2.99)	0.74	0.49 (0.09-2.47)	0.39
	**Decreased ovarian reserve**	1.37 (0.48-3.91)	0.55	0.86 (0.35-2.13)	0.76
	**Mixed**	1.03 (0.49-2.17)	0.93	1.16 (0.59-2.26)	0.66
	**Unexplained**	0.93 (0.40-2.19)	0.88	1.24 (0.57-2.69)	0.57
**Fertilization method**					
* *	**ICSI** * *	Ref	Ref	
	**IVF**	0.42 (0.24-0.76)	< 0.01	1.32 (0.74-2.35)	0.33
	**Mixed**	0.64 (0.27-1.53)	0.32	0.61 (0.23-1.59)	0.32
**Group**					
* *	**A** * *	Ref	Ref	
	**B**	0.75 (0.33-1.68)	0.50	1.47 (0.67-3.19)	0.33
	**C**	1.33 (0.47-3.71)	0.59	1.06 (0.41-2.70)	0.90
**Transfer (fresh vs. FET)**		1.27 (0.72-2.26)	0.40	0.85 (0.50-1.44)	0.55
**Ovarian stimulation protocol**					
* *	**GnRH antagonist** * *	Ref	Ref	
	**GnRH agonist**	0.69 (0.31-1.52)	0.36	1.49 (0.71-3.13)	0.29
	**Microdose flare**	1.14 (0.39-3.31)	0.80	1.58 (0.71-3.54)	0.26
**AMH (ng/ml) ** * *		0.94 (0.86-1.03)	0.21	1.02 (0.93-1.11)	0.65
**Estradiol on the day of trigger (pg/ml) **		1.00 (1.00-1.00)	0.52	1.00 (1.00-1.00)	0.65
**Number of retrieved oocytes**		1.06 (0.94-1.20)	0.29	0.98 (0.89-1.08)	0.75
**Number of MII oocytes**		0.91 (0.79-1.04)	0.17	1.02 (0.92-1.15)	0.62
**Number of 2PNs**		1.02 (0.94-1.11)	0.55	1.02 (0.94-1.11)	0.55
**Endometrial thickness (mm)**		1.09 (0.94-1.26)	0.24	0.89 (0.77-1.02)	0.11
**Newborn gender (male vs. female)**		0.96 (0.57-1.60)	0.88	1.30 (0.81-2.09)	0.27
**Method of delivery (NVD vs. C/S)**		0.44 (0.23-0.82)	0.01	2.18 (1.18-4.02)	0.01
Model including all variables in the first column, Group A: Single embryo transfer using a good-quality embryo, Group B: Double embryo transfer using two good-quality embryos, Group C: Double embryo transfer using one good-quality embryo plus one poor-quality embryo, NVD: Normal vaginal delivery, C/S: Cesarean section, FET: Frozen-thawed embryo transfer, BMI: Body mass index, PCOS: Polycystic ovarian syndrome, IVF: In vitro fertilization, ICSI: Intracytoplasmic sperm injection, GnRH: Gonadotropin releasing hormone, AMH: Anti-Mullerian hormone, MII: Metaphase II, 2PN: 2 pronuclear, OR: Odds ratio, CI: Confidence interval					

## 4. Discussion

Our results showed that neonates born after SET of a cleavage-stage GQE had a similar birth weight compared to those born after DET of two GQEs or DET of a GQE plus a PQE embryo during both fresh and FET cycles. Therefore, our research hypothesis that the addition of a PQE to a GQE, when transferred together, may decrease the neonate's birth weight was rejected.

Previously, the association between embryo quality and pregnancy outcome had been investigated in several studies and the results showed that transfer of PQEs could adversely affect clinical pregnancy and live birth (10-12). Throughout recent years it has been stated that this adverse effect may not be limited to live birth, and may in fact extend to neonatal outcomes such as birth weight (2). The earlier studies evaluated the effect of embryo quality on perinatal outcomes in fresh cycles and reported that the transfer of either GQEs or PQEs in the cleavage or blastocyst stages did not affect the neonatal birth weight, or the risk of PTB or being small for gestational age (13-15).

On the other hand, with improvement in ART, the transfer of frozen-thawed embryos has become an option for women undergoing IVF, especially for those who are at risk of developing ovarian hyperstimulation syndrome. However, it has been shown that freezing-thawing procedures can negatively affect perinatal outcomes of singleton pregnancies (5). Regarding birth weight, it has been claimed that there is a strong correlation between embryo cryopreservation and birth weight variations (4). Two studies involving FET cycles compared neonatal outcomes after transferring PQEs or GQEs. They found decreased birth weight among neonates born following the transfer of PQEs in comparison with GQE transfer, regardless of cleavage or blastocyst stage (2, 16).

All of the above-mentioned studies compared the perinatal outcomes following the transfer of either PQEs or GQEs. Only five studies have examined the impact of adding one PQE to a GQE on ART outcomes and two of these investigated the effect of transferring a PQE and a GQE together on neonatal birth weight (17-21). Similar to our findings, Li and colleagues examined fresh cycles in which one cleavage-stage PQE plus one GQE were transferred, and compared these cycles with those in which two GQEs were transferred, in terms of ART outcomes. They did not find any significant differences regarding pregnancy, miscarriage or live birth rates between the two groups. Moreover, according to their results, an additional PQE did not negatively affect the neonatal birth weight, gestational age or risk of PTB (19). Likewise, another recent study reached the same conclusion. The study evaluated ART outcomes among women who received either DET with a PQE and a GQE or SET with only a GQE during FET of blastocysts. The results showed that the neonatal birth weight was similar between both groups. However, the women in the double blastocyst transfer group comprising one PQE plus one GQE achieved significantly higher pregnancy and live birth rates (20). In line with the two aforementioned studies, our findings indicated that adding a PQE to a GQE did not adversely affect preterm birth or birth weight either in fresh or in FET cycles.

The mechanism by which embryo quality could influence birth weight remains unknown. It has been proposed that epigenetic changes during the culture period may be involved in this process and could affect fetal growth (22). Moreover, the methylation levels and any altered homeostasis or impaired metabolism may have consequences for normal fetal growth and could affect neonatal birth weight (2, 19).

It should be noted that DET with a GQE accompanied by a PQE usually means that there were no more GQEs for transfer and therefore the transferred GQE had to have come from a poor cohort. Theoretically, a cohort of PQEs may negatively affect the GQE potential during the co-culture period. However, the adverse effect of the PQE on the GQE, when those two are transferred together in a DET procedure, is highly debatable. Published data have suggested that embryo growth and development can be positively affected by group culturing (17, 23).

Nevertheless, it has been indicated that the presence of PQEs in the culture medium may have a detrimental effect on the developmental process of GQEs (24). On the other hand, the evidence shows that the endometrium plays a selective role and prevents PQEs from implantation (17). It should also be noted that morphology is not the only predictor for embryo quality and other factors such as embryo ploidy status or mitochondrial issues should be considered.

Other studies have focused on either fresh or FET cycles; however, for the first time in this study we compared the effect of adding a PQE to a good-quality one on neonatal birth weight between fresh and FET cycles. It should be noted that twin pregnancies and other pregnancy-linked problems triggering intrauterine fetal growth retardation such as gestational diabetes, pregnancy-induced hypertension and preeclampsia were excluded from the study. In addition, parental basic characteristics associated with embryo quality and neonatal outcomes including age, BMI and poor ovarian reserve were considered for further analysis. However, our results did not change after adjusting for these confounding variables.

The main limitation of this study was its retrospective design. The data were extracted from electronic medical records with some missing information. Patients' follow-up was done using a phone questionnaire, which was less accurate than the medical records. Another limitation may be the subjectivity in the embryo grading process. However, the procedure was performed in a single center where embryos were graded by the same group of trained embryologists. The study was also limited by the small sample size, preventing more subgroup analysis.

## 5. Conclusion

In conclusion, our results showed that a PQE did not have a significant influence on a GQE regarding neonatal birth weight when transferred together. This study was a retrospective one and naturally a randomized controlled trial would be more persuasive.

##  Conflict of Interest

The authors declare that there is no conflict of interest.
